# Adaptive DBS at Match Point: Can it Make Mistakes and Still be Successful?

**DOI:** 10.1002/mdc3.70479

**Published:** 2025-12-22

**Authors:** Mario Sousa, Gerd Tinkhauser

**Affiliations:** ^1^ Department of Neurology Bern University Hospital and University of Bern Bern Switzerland

**Keywords:** adaptive DBS, artifacts, closed‐loop DBS, Parkinson's disease

We read with great interest the response of our esteemed colleagues, Müller and Contarino^1^, to our recent letter on neuromodulation.^2^ In our original report, we described a patient exhibiting severe ECG artifacts in local field potential (LFP) recordings, likely attributable to the implantation of the neurostimulator in the left chest, with the implantation site chosen to accommodate the patient's desire for an unrestricted forehand tennis swing. In contrast, Müller and Contarino present a patient with a right‐sided implant who successfully resumed tennis after Deep Brain Stimulation (DBS). Notably, chronic LFP recordings in this patient revealed intermittent increases in LFP power that coincided temporally with periods on the tennis court. Together, these complementary cases highlight two key technical limitations of current adaptive DBS systems, cardiac artifacts and movement‐related artifacts. As enthusiastic supporters of both adaptive DBS and tennis, we could not resist continuing this exchange into a friendly “third set.” Here, we would like to introduce and emphasize the concept of *error tolerance* in adaptive DBS. Adaptive DBS algorithms interact with feedback signals exactly as programmed. However, when those input signals are corrupted by artifacts, the resulting control output may diverge from the intended modulation of pathological brain circuits. From a neuroscientific perspective this is frustrating, but from a clinical perspective, the relevance of such deviations may depend on their nature. While some artifacts can continuously obscure clinically relevant information, thereby compromising the algorithm around the clock. Others can occur only in specific situations, as for example during large‐scale movements, hereby introducing episodic adaptive control errors. The ideal scenario with adaptive stimulation perfectly and meaningfully matching the patient's underlying neurophysiology at all times is probably unattainable in reality. Not only because of current technical limitations, but also because no neurophysiological biomarker accounts for 100% of clinical or pharmacological variance. Therefore, the need of an *error tolerance* is inevitable. Accordingly, we must understand and define appropriate margins for algorithmic error and ensure patients’ safety and comfort during brief periods of unintentional stimulation at the upper or lower bounds of the programmed control range.

Returning to tennis: in 2025, Jannik Sinner achieved an exceptional 58–6 win–loss record (a 90.6% match–win rate) despite winning only 56% of total points played. This demonstrates that top tennis players barely win more than half of the points they play, yet still achieve outstanding overall outcomes. Similarly, in adaptive DBS, we do not yet know how consistently the therapy must align with patients’ state for it to be perceived as superior to conventional DBS. Identifying the acceptable margin of error, as well as individual factors influencing tolerance to brief periods of suboptimal stimulation, will be essential for guiding the development and programming of adaptive DBS.

## Author Roles

(1) Research project: A. Conception, B. Organization, C. Execution; (2) Statistical Analysis: not applicable; (3) Manuscript Preparation: A. Writing of the first draft, B. Review and Critique.

M.S.: 1A, 1B, 1C, 3A, 3B

G.T.: 1A, 1B, 1C, 3A, 3B

## Disclosures

**Ethical Compliance Statement:** The authors confirm that the approval of an institutional review board was not required for this work, and as no individual patient information is presented, informed consent was not required. We confirm that we have read the Journal's position on issues involved in ethical publication and affirm that this work is consistent with those guidelines.

**Funding Sources and Conflict of Interest:** GT has a research agreement with RuneLab, he also receives financial support from Medtronic, Boston and Spirig, not related to the present work; MS receives financial support from Boston Scientific, Medtronic, Zambon and Bial not related to the present work.

**Financial Disclosures and Conflicts of Interest:** GT receives funding from the Swiss National Science Foundation (project number: PZ00P3_202166) and the Swiss Parkinson Association; MS receives funding from Gottfried und Julia Bangerter‐Rhyner‐Stiftung.

## Data Availability

Data sharing not applicable to this article as no datasets were generated or analysed during the current study.
